# Validity and reliability of the sleep health index among community-dwelling older adults

**DOI:** 10.3389/fpubh.2026.1651175

**Published:** 2026-04-22

**Authors:** Miao Miao, Nan Zhang, Bingqian Zhu, Jingyao Yu, Ying Chen, Subiyinuer Maimaiti, Ping Yan

**Affiliations:** 1 School of Nursing, Xinjiang Medical University, Urumqi, China; 2 Xiangya School of Nursing, Central South University, Changsha, China; 3 Health Care Research Center for the Xinjiang Regional Population, Xinjiang Medical University, Urumqi, China; 4 School of Nursing, Shanghai Jiao Tong University, Shanghai, China; 5 School of Health Management, Xinjiang Medical University, Urumqi, China

**Keywords:** older adult, reliability, sleep health, sleep quality, validity

## Abstract

**Background:**

Sleep health represents a positive and multidimensional framework that extends beyond the absence of sleep disturbances or insufficient sleep duration. Despite its clinical relevance, empirical research on sleep health in older adults remains limited. The Sleep Health Index (SHI) was developed to comprehensively evaluate sleep health. This study aimed to validate the Chinese version of the SHI among community-dwelling older adults.

**Methods:**

This is a cross-sectional design. Older adults aged 60 years and above were recruited (*n* = 274), and a subset (*n* = 60) was invited to complete the test–retest survey after a two-month interval. The SHI-C, Pittsburgh Sleep Quality Index (PSQI), Insomnia Severity Index (ISI), Epworth Sleepiness Scale (ESS), 15-Item Geriatric Depression Scale (GDS-15), comorbidity burden (aCCI), and handgrip strength (HGS) were administered to assess sleep health and related health indicators. The SHI-C was evaluated for structural, convergent, concurrent, known-group validity, internal consistency, and test–retest reliability.

**Results:**

Exploratory factor analysis supported a three-factor structure, which was subsequently confirmed by confirmatory factor analysis (i.e., sleep quality, sleep duration, and disordered sleep), consistent with the original validation study. Parallel analysis further confirmed the three-factor solution. Measurement invariance testing across gender supported full metric and scalar invariance. The SHI-C demonstrated concurrent validity through strong negative correlations with both the PSQI (*r* = −0.624) and ISI (*r* = −0.696). It also showed weak but significant associations with the ESS (*r* = −0.328) and the GDS-15 (*r* = −0.329). Known-group validity was supported as participants with chronic diseases, insomnia, or depression had significantly lower SHI-C scores (*p* < 0.001). Furthermore, the total score of SHI-C exhibited a significant negative correlation with the aCCI (*r* = −0.302) and a significant positive correlation with HGS (*r* = 0.171), supporting its association with key objective health indicators. The SHI-C showed acceptable internal consistency (Cronbach’s *α* = 0.775) and test–retest reliability (intraclass correlation coefficient = 0.784).

**Conclusion:**

The SHI-C is a reliable and valid measurement for assessing sleep health in community-dwelling older adults. These findings suggest its potential utility for the sleep health assessment among this population, thereby contributing to the geriatric care enhancement.

## 1 Introduction

The global population is experiencing irreversible aging, with Chinese older adult population projected to reach 366 million by 2050, accounting for 26.10% of the total population (1). This demographic shift presents significant public health challenges. Quality sleep is essential for overall health. Epidemiological studies indicate a high prevalence of sleep disturbances among older adults in China, ranging from 33.80% in rural communities to 67.30% among nursing home residents (2–4). Sleep health is a critical determinant of healthy aging. It represents a positive, multidimensional framework for conceptualizing and assessing individuals’ sleep, moving beyond a sole focus on sleep disturbances or disorders. It is also associated with higher cardiovascular risks (5), metabolic dysregulation (6), and cognitive impairment (7). Sleep health is defined as a multidimensional pattern of sleep-wakefulness adapted to individual, social, and environmental demands, promoting physical and mental well-being (8). This definition aligns with the WHO’s positive health view, framing sleep as an adaptive process interacting with context and offering a holistic framework beyond biological metrics. Accumulating evidence demonstrates that sleep health is a critical indicator that can provide concrete targets for health promotion and prevention activities. However, given the holistic definition of sleep health, its measurement has become a challenge.

To address these challenges, previous studies have developed different measurements to assess sleep health. Besides them, the RU-SATED framework (8) and the Sleep Health Index (SHI) (9) are two commonly used instruments. The RU-SATED framework comprises 6 items, covering six aspects of sleep health, and has been translated into multiple languages. However, significant differences in internal consistency and factor structure have been observed across different versions (8, 10–12). The SHI was developed by the National Sleep Foundation (NSF), and includes 14 items that comprehensively address key dimensions of sleep health, comprising sleep quality, sleep duration, and disordered sleep. Prior studies have demonstrated that good internal consistency of the SHI across various language versions (Cronbach’s *α* ranging from 0.67 to 0.75) (9, 13). Researchers have conducted the cross-cultural adaptation and validation of the Chinese version of the SHI (SHI-C), and tested it in pregnant women and patients with spinal degenerative diseases (14–16). They demonstrated that the SHI-C exhibited acceptable validity and reliability across various Chinese populations. However, the unique physiological and psychosocial factors affecting sleep in older adults, particularly age-related circadian rhythm shifts, prevalence of multimorbidity, and social isolation patterns, necessitate population-specific validation. Given the current absence of psychometric data for the SHI-C in this demographic, establishing measurement equivalence becomes critical for addressing sleep-related health disparities in aging societies.

Evidence suggests that certain populations, such as older adults from socioeconomically disadvantaged backgrounds, are more susceptible to sleep health disparities (SHDs) (17, 18), a term referring to systematic differences in one or more dimensions of sleep health that adversely affect designated disadvantaged groups. Given this, it is crucial to measure sleep health accurately within diverse population groups. This study focuses specifically on older adults with distinct health risk profiles. The objective is to assess the validity and reliability of the SHI-C among community-dwelling older adults. Our findings may provide more evidence for selecting appropriate tools for older adults with varying health profiles, to broaden the application of the SHI-C, and thus deepen the understanding of SHDs in aging populations.

## 2 Methods

### 2.1 Study design

This study was conducted as part of a large-scale epidemiological parent survey entitled “The Comprehensive Demonstration and Application of Risk Warning and Prevention and Control Technologies for Geriatric Syndromes in Xinjiang.” The parent study employed a cross-sectional design, implemented in community-based primary healthcare centers in Xinjiang Uyghur Autonomous Region, China, from August to September 2024. Its overarching aims were to investigate the prevalence and associated factors of common geriatric syndromes and to inform risk assessment and preventive strategies for the local older population. Ethical approval was obtained from the Research Ethics Committee of Xinjiang Medical University (IRB code: XJYKDXR20220725029).

### 2.2 Participants

Xinjiang is a multi-ethnic settlement area, predominantly composed of the Han and Uyghur ethnic groups (together accounting for 87.20% of the total population) (19). To capture diversity in residence (urban/rural) and health status (such as severe chronic diseases and cognitive impairment), a community-based convenience sampling strategy was employed to enrich the sample profile. To maximize community coverage, participating community health service centers were responsible for notifying and recruiting the entire older population within their catchment areas. Given the vast territory of Xinjiang and the notable differences in lifestyles, sleep patterns, and health profiles between the populations in the north and south of Xinjiang, geographically separated by the Tianshan Mountains, we selected two distinct sites for sample recruitment as Shache County in southern Xinjiang and Yining County in northern Xinjiang. The inclusion criteria were: (1) aged 60 years and above; (2) language proficiency: ensure accurate questionnaire understanding; (3) being conscious and capable of cooperating to complete the interview and health examinations. Participants who refused to sign the consent form were excluded from the study. A total of 293 participants were recruited and 19 were excluded due to invalid responses (e.g., responses with contradictions or a clear pattern), resulting in 274 participants included in the final analysis. For the test–retest reliability analysis, a subset (*n* = 60) of the original participants was randomly selected to complete the SHI-C again using a two-month interval (20).

### 2.3 Data collection

Participants were recruited with the help of administrators and healthcare workers in local primary healthcare settings. Informed consent was obtained prior to data collection. Trained investigators conducted face-to-face interviews to administer questionnaires and perform health checks, thereby ensuring the accuracy and completeness of the collected data. Participants who agreed to take part in the retest were invited for a second face-to-face interview to complete the SHI-C again. Each participant will receive a compensation of 10 RMB after complete the survey.

### 2.4 Sample size

A total of 274 participants were included in this study. The sample size was determined based on the common rule for factor analysis, which recommends a minimum of 200 participants or 5–10 cases per variable (21). With 14 items in the SHI, our sample exceeds these requirements. Notably, our sample size is consistent with those reported in two prior validation studies by Mu et al. in Chinese population, which enrolled 271 college students and 265 patients with spinal degenerative diseases were enrolled for analyzes, respectively (14, 16).

### 2.5 Measurements

#### 2.5.1 Sociodemographic survey

A Sociodemographic survey was used to measure demographics and health-related characteristics, including age, gender, education level, marital status, and health status (chronic diseases, body mass index, vision, hearing, self-rated health status).

#### 2.5.2 Sleep health index (SHI)

The SHI is a widely utilized instrument for assessing sleep health, originally validated through nationally representative samples of US adults (9). This scale consisted of 14 items across three domains: sleep duration, sleep quality, and disordered sleep. Each domain score ranges from 0 to 100, with the total score calculated as the mean of the three domain scores, where higher scores indicate better sleep health. In the original study, the SHI demonstrated acceptable internal consistency (Cronbach’s *α* of 0.75 for the overall index, 0.63 for sleep duration and disordered sleep, and 0.77 for sleep quality) (9). Building upon this framework, Mu et al. (14) completed the cross-cultural adaptation and validation of the Chinese version (SHI-C) in 2023. Through testing across diverse Chinese adult populations including college students, pregnant women, and patients with spinal degenerative diseases (14–16), the SHI-C demonstrated satisfactory psychometric properties. Among these, the overall Cronbach’s *α* was 0.73 in the sample of college students (14). Empirical studies have confirmed the SHI-C as a valid tool for evaluating sleep health status in Chinese populations.

#### 2.5.3 Pittsburgh sleep quality index (PSQI)

The PSQI is used to measure overall sleep quality over the past month (22). It consists of 19 self-rated items across seven components: subjective sleep quality, sleep latency, sleep duration, habitual sleep efficiency, sleep disturbances, use of sleeping medication, and daytime dysfunction. Each item is scored from 0 to 3, with the global score ranging from 0 to 21. Higher scores indicate worse sleep quality, with a global score over 5 indicating sleep disturbance. The Cronbach’s *α* coefficient of the Chinese version of PSQI among older adults was 0.68 in a previous study (23). The Cronbach’s α of PSQI in this study was 0.65.

#### 2.5.4 Insomnia severity index (ISI)

The ISI is a 7-item questionnaire assessing the nature, severity, and impact of insomnia symptoms during the past 2 weeks (24), and was used to measure insomnia in this study. A 5-point Likert scale is used to rate each item, a total score ranges from 0 to 28, with higher scores indicating more severe insomnia symptoms. A total score of 10 or above indicates the presence of insomnia (24, 25). The Chinese version of ISI demonstrated a good internal consistency in a previous study (Cronbach’s *α* = 0.81) (26). The Cronbach’s α of ISI in this sample was 0.88.

#### 2.5.5 Epworth sleepiness scale (ESS)

The ESS was used to measure average daytime sleepiness (27). Participants rated their likelihood of dozing on a 4-point Likert scale in eight different daily situations. The total score ranges from 0 to 24, with higher scores indicating higher levels of daytime sleepiness. A score above 10 indicates excessive daytime sleepiness. The Cronbach’s *α* of the Chinese version of ESS was 0.81 in previous study (28) and 0.79 in this study.

#### 2.5.6 15-item geriatric depression scale (GDS-15)

The GDS-15 was used to measure the level of depressive symptoms (29). Participants answered “yes” or “no” to 15 questions depending on how they had felt over the past week. A total score of 0–4 indicates normal mood or emotional health status, 5–9 indicates mild depression, and 10–15 indicates moderate to severe depression. The Chinese version of GDS-15 demonstrated good internal consistency in a previous validation study (Cronbach’s *α* = 0.81) (30). The Cronbach’s α of GDS-15 in this sample was 0.76.

#### 2.5.7 Age-adjusted Charlson comorbidity index (aCCI)

The aCCI was used to assess comorbidity burden in participants aged ≥60 years (31). The score combines the weighted points (1–6) from 19 conditions with an age adjustment of 2 points (60–69 years), 3 points (70–79 years), or 4 points (≥80 years), consistent with adding one point per decade above age 50. The total aCCI score represents the sum of condition weights and age-adjusted points (32).

#### 2.5.8 Handgrip strength (HGS)

HGS was assessed using a digital hand dynamometer (model EH101; Camry Electronic Company, Xiangshan, Guangdong, China). Participants performed three trials with their dominant hand, with a rest interval of at least 15 s between trials. The maximum value (in kg) was recorded for analysis.

### 2.6 Statistical analysis

Statistical analyzes were conducted using SPSS 25.0 (IBM, Armonk, NY, USA) and Mplus 8.3 (Muthén & Muthén, Los Angeles, CA, USA). Descriptive statistics were employed to summarize participant characteristics. Categorical variables were presented as frequency and percentage, and continuous variables as mean with standard deviation (SD). To ensure the accuracy and reliability of the results, we employed multiple methods to handle missing data. For variables with ≤ 5% missing data, imputation using the mean for normally distributed data or median was performed. Variables with > 5% missing data were excluded from subsequent analyses to preserve data integrity (33).

To examine the underlying factor structure of the scale, an Exploratory Factor Analysis (EFA) was first conducted using Principal Axis Factoring with Promax oblique rotation. Sampling adequacy was evaluated using the Kaiser-Meyer-Olkin (KMO) measure and Bartlett’s test of sphericity. Data were considered suitable for factor analysis if KMO > 0.70 and Bartlett’s test was significant (*p* < 0.05) (34). The number of factors was determined by Horn’s parallel analysis based on 100 random data simulations, supplemented by the eigenvalue > 1 criterion. Factors were retained when their empirical eigenvalues exceeded the 95th percentile of the corresponding eigenvalues obtained from the random data (35). Given the prior theoretical and empirical evidence supporting a three-factor structure (14), the EFA was conducted as a preliminary examination of the item-factor configuration rather than as a purely data-driven exploration. Confirmatory factor analysis (CFA) was subsequently performed to test the hypothesized three-factor structure identified by the EFA (36). Both EFA and CFA were conducted using the total sample. Model fit in CFA was evaluated using the chi-square to degrees of freedom ratio (χ^2^/df), root mean square error of approximation (RMSEA), comparative fit index (CFI), goodness-of-fit index (GFI), and Tucker-Lewis index (TLI). Model fit was considered acceptable if χ^2^/df < 3, RMSEA < 0.08, CFI > 0.90, GFI > 0.90, and TLI > 0.90 (37). Furthermore, multi-group measurement invariance across gender was assessed using a nested model approach to examine configural, metric, and scalar invariance, employing robust maximum likelihood estimation. Measurement invariance was supported if the chi-square difference test between successive models was non-significant (*p* > 0.05) (38).

Concurrent validity was assessed by correlating SHI-C scores with scores from other validated instruments, including the PSQI, ESS, and ISI. Convergent validity was assessed by correlating SHI-C scores with scores measuring related constructs (e.g., sleepiness, quality of life, and depression), such as the ESS, and GDS-15. The associations were examined using Pearson or Spearman correlation analyzes, with the following criteria: *r* < 0.30 (weak correlation), 0.30 ≤ *r* ≤ 0.60 (moderate correlation), and *r* > 0.60 (strong correlation) (39). Known-group validity was assessed by comparing SHI-C scores among different groups (e.g., gender, depression, chronic disease, poor sleep quality, and insomnia) using independent samples t-test or one-way analysis of variance. Statistical significance was set at *p* < 0.05 (two-tailed).

Cronbach’s *α* was calculated to assess the internal consistency of the SHI-C, with values higher than 0.7 considered acceptable (40). Test–retest reliability was assessed using the intraclass correlation coefficient (ICC) with the following criteria: ICC < 0.40 (poor); 0.40 ≤ ICC ≤ 0.59 (average), 0.60 ≤ ICC ≤ 0.74 (acceptable) and ICC > 0.75 (good) (41). ICC was calculated by the two-way mixed effects model, single measurement type, and absolute agreement (42). Statistical significance was set at *p* < 0.05 (two-tailed).

## 3 Results

### 3.1 Baseline characteristics

Participant characteristics at baseline are summarized in Table 1. The mean age of the participants was 67.61 years (SD 5.84, range 60–89), and 48.20% of participants were males. A majority of the participants suffered from chronic diseases (71.50%). The mean total score of the SHI-C was 84.29 (12.58). The sub-index scores for sleep duration, sleep quality, and disordered sleep were 91.43 (11.98), 70.18 (21.98), and 91.30 (19.39), respectively. Based on the PSQI and the ISI, 72.30% of participants were classified as having poor sleep quality, and 24.50% met criteria for insomnia. Additionally, 23.10% of the participants were diagnosed with depression. Regarding key health and functional indicators, the mean aCCI was 3.85 (2.35), and the mean HGS was 18.58 (7.64) kg.

**Table 1:** Demographic and clinical characteristics of the study participants (*n* = 274).

Variables	Mean (SD) or *n* (%)	Range
Age (years)	67.61 (5.84)	60–89
Sex (male)	132 (48.18%)	
Education		
Elementary school and below	198 (72.26%)	
Middle school	63 (23.00%)	
High school and above	13 (4.74%)	
Marital status		
Married	174 (63.50%)	
Single / Divorce / Separation	100 (36.50%)	
Chronic diseases		
Yes	196 (71.53%)	
No	78 (28.47%)	
BMI (kg / m^2^)		
Overweight (BMI ≥ 23 kg / m^2^)	196 (71.53%)	17-48
Vision		
Decline	94 (34.31%)	
Normal	180 (65.69%)	
Hearing		
Decline	84 (30.66%)	
Normal	190 (69.34%)	
Self-assessed health status		
Good	94 (34.31%)	
Average	120 (43.79%)	
Poor	60 (21.90%)	
SHI-C	84.29 (12.58)	42–99
Sleep duration	91.43 (11.98)	43–100
Sleep quality	70.18 (21.98)	5–100
Disordered sleep	91.30 (19.39)	29–100
PSQI	8.10 (3.98)	1–21
Poor sleep quality (PSQI > 5)	198 (72.26%)	
ISI	6.51 (5.14)	0–23
Insomnia (ISI > 9)	67 (24.45%)	
ESS	2.87 (3.34)	0–16
GDS-15	2.78 (2.53)	0–12
15-Item Geriatric Depression Scale (GDS-15 ≥ 5)	63 (23.99%)	
aCCI	3.85 (2.35)	0–12
HGS (kg)	18.58 (7.64)	2–49

SHI-C, The Chinese Sleep Health Index; PSQI, Pittsburgh Sleep Quality Index; ISI, Insomnia Severity Index; ESS, Epworth Sleepiness Scale; GDS-15, 15-Item Geriatric Depression Scale; aCCI, Age-adjusted Charlson Comorbidity Index; HGS, Handgrip Strength.

### 3.2 Validity

#### 3.2.1 Structural validity

The KMO measure indicated sampling adequacy (KMO = 0.774), and Bartlett’s test of sphericity was significant (*χ*^2^ = 1047.569, *p* < 0.001), supporting the suitability of the data for factor analysis. EFA using principal axis factoring with Promax rotation identified a three-factor structure of the SHI-C among older adults. Parallel analysis based on 100 random datasets further supported the retention of three factors, which was aligned with the hypothesized structure of the SHI-C. CFA of the three-factor model demonstrated an acceptable model fit, with *χ*^2^/df = 1.83, RMSEA = 0.055, CFI = 0.954, TLI = 0.936, and SRMR = 0.054. The factor structure of the SHI-C is presented in Table 2. The majority of items demonstrated substantial standardized factor loadings exceeding 0.400. However, the “sleep medication” item in the disordered sleep sub-index exhibited a low loading of 0.150, and the “unintentional doze” item in the sleep quality sub-index also showed a relatively low loading (0.216). Measurement invariance across gender was examined using multigroup CFA. The results supported full metric and scalar invariance, with non-significant chi-square difference tests between the configural and metric models (*p* = 0.127) and between the metric and scalar models (*p* = 0.580).

**Table 2:** Confirmatory factor analysis results for the scale items (*n* = 274).

Factors	Items	Standardized regression weights
Sleep quality	Sleep quality rating	0.480
	Well-rested	0.889
	Trouble falling asleep	0.900
	Trouble staying asleep	0.730
	Negative impact	0.639
	Unintentional doze	0.216
Disordered sleep	Sleep medication	0.150
	Diagnosed sleep disorder	0.633
	Sleep doctor	0.852
Sleep duration	Weekday sleep	0.666
	Sleep deficit	0.793
	Sleep variability	0.426

#### 3.2.2 Concurrent, convergent, and criterion-related validity

Table 3 presents correlations of SHI-C with different measures. The overall SHI-C was correlated highly with PSQI and ISI (*r* = −0.624 and −0.696). The sleep quality sub-index was correlated highly with PSQI and ISI (*r* = −0.728 and −0.767). The disordered sleep sub-index showed a moderate correlation with ISI (*r* = −0.442), and the sleep quality sub-index with GDS-15 (*r* = −0.437). Both sub-indices were correlated weakly with ESS (*r* = −0.228 and −0.289). In addition, the aggregate SHI-C exhibits a notable correlation with ESS, and GDS-15 with correlation coefficients of −0.328 and −0.329, respectively. Regarding the correlations of the SHI-C with key objective health criteria, the overall SHI-C score showed a significant negative correlation with the aCCI (*r* = −0.302) and a significant positive correlation with HGS (*r* = 0.171).

**Table 3:** Bivariate correlations among the study variables (*n* = 274).

Variables	Overall SHI-C	Sleep duration	Sleep quality	Disordered sleep
Sleep duration	0.383**			
Sleep quality	0.800**	−0.003		
Disordered sleep	0.802**	0.133*	0.423**	
PSQI	−0.624**	0.022	−0.728**	−0.401**
ISI	−0.696**	−0.063	−0.767**	−0.442**
ESS	−0.328**	−0.130*	−0.289**	−0.228**
GDS-15	−0.329**	0.076	−0.437**	−0.189**
aCCI	−0.302**	−0.033	−0.243**	−0.288**
HGS	0.171**	−0.111	0.210**	0.166**

**p < 0.001, *p < 0.05; SHI-C, The Chinese Sleep Health Index; PSQI, Pittsburgh Sleep Quality Index; ISI, Insomnia Severity Index; ESS, Epworth Sleepiness Scale; GDS-15, Geriatric Depression Scale-15 Item; aCCI, Age-adjusted Charlson Comorbidity Index; HGS, Handgrip Strength.

#### 3.2.3 Known-group validity

The SHI-C scores in different groups are shown in Table 4. No significant differences were found across age groups, educational levels, or marital status. However, male participants had significantly higher overall SHI-C and sleep quality sub-index scores compared to females (*p* < 0.05). Noteworthy, participants with chronic diseases had significantly lower scores on the sleep quality sub-index than those without (66.08 *vs.* 80.50). Those with depression, poor sleep quality, and insomnia had lower scores on the overall SHI-C and all three sub-indices than those without (*p* < 0.05).

**Table 4:** Comparison of SHI-C scores across demographic subgroups (*n* = 274).

Variables	Overall SHI-C	Sleep duration	Sleep quality	Disordered sleep
Age				
60 ~ 69	84.15 (12.67)	90.98 (12.39)	69.60 (22.33)	91.87 (19.37)
70 ~ 79	85.10 (11.65)	92.07 (11.24)	72.27 (20.39)	91.11 (18.06)
≥80	81.58 (16.91)	94.50 (9.62)	66.67 (36.30)	83.33 (26.67)
Sex				
Male	86.04 (11.80)*	90.21 (12.44)	74.84 (20.28)**	93.05 (17.65)
Female	82.67 (13.10)	92.56 (11.46)	65.85 (22.67)	89.66 (20.80)
Education				
Elementary school and below	84.83 (12.59)	91.68 (11.71)*	70.68 (22.78)	92.17 (18.80)
Middle school	83.05 (12.83)	92.06 (11.10)*	67.68 (21.20)	89.40 (19.88)
High school and above	82.08 (11.44)	84.54 (17.93)*	74.69 (9.49)	87.15 (25.69)
Marital status				
Married	84.91 (11.90)	91.33(12.01)	70.93 (21.46)	92.44 (17.82)
Single / Divorce / Separation	83.22 (13.68)	91.60(11.98)	68.88 (22.90)	89.30 (21.80)
Chronic diseases				
Yes	82.23 (12.90)**	91.65 (11.91)	66.08 (21.59)**	89.02 (21.19)**
No	89.46 (10.10)	90.87 (12.21)	80.50 (19.51)	97.01 (12.25)
Poor sleep quality (PSQI > 5)				
Yes	80.97 (12.48)**	91.60 (12.16)	63.05 (20.00)**	88.29 (21.58)**
No	92.95 (7.86)	90.97 (11.55)	88.76 (14.94)	99.12 (7.69)
Insomnia (ISI > 9)				
Yes	71.27 (13.25)**	90.54 (12.68)	45.34 (15.47)**	78.04 (27.75)**
No	88.51 (8.97)	91.71 (11.76)	78.22 (17.25)	95.58 (13.26)
Depression (GDS-15 ≥ 5)				
Yes	77.27 (13.44)**	92.60 (12.18)	55.03 (19.46)**	84.30 (25.09)**
No	86.39 (11.54)	91.08 (11.92)	74.71 (20.65)	93.38 (16.85)

**p < 0.001, *p < 0.05; SHI-C, The Chinese Sleep Health Index; PSQI, Pittsburgh Sleep Quality Index; ISI, Insomnia Severity Index; GDS-15, Geriatric Depression Scale-15 Item.

### 3.3 Reliability

The reliability data are presented in Table 5. The Cronbach’s *α* for the overall SHI was 0.775. For the sub-indices, Cronbach’s α values were 0.636 for sleep duration, 0.825 for sleep quality, and 0.515 for disordered sleep, respectively. Regarding test–retest reliability, the ICC were 0.536 for sleep duration, 0.857 for sleep quality, and 0.728 for disordered sleep, respectively.

**Table 5:** Reliability analysis of the SHI-C scale (*n* = 274).

Factors	Reliability	
	Internal consistency (*n* = 274, Cronbach’s α)	Test–retest reliability (*n* = 60, ICC)
Overall SHI-C	0.775	0.784
Sleep duration	0.636	0.536
Sleep quality	0.825	0.857
Disordered sleep	0.515	0.728

**p < 0.001; SHI-C, The Chinese Sleep Health Index; ICC, intraclass correlation coefficient.

## 4 Discussion

To the best of our knowledge, this is the first study to validate the SHI-C in community-dwelling older adults. Confirmatory factor analysis supported the scale’s original three-factor structure. The SHI-C demonstrated robust construct validity, evidenced by its expected correlations with established measures of sleep, mood, and daytime function, its ability to distinguish individuals by chronic disease status, and its significant associations with objective health indicators. Reliability was generally acceptable, with satisfactory internal consistency for the overall scale and good test–retest reliability, although the disordered sleep subscale showed modest internal consistency and a ceiling effect. Overall, these findings support the utility of the SHI-C for assessing multidimensional sleep health in older adults, while also highlighting areas for potential refinement in this specific demographic.

In this study, the total SHI-C score was relatively higher than reports from other populations and areas (9, 13, 16, 43), but slightly lower than those reported for Chinese college students and pregnant women (14, 15). Notably, the sleep quality domain had the lowest score among the three SHI-C dimensions, suggesting that a substantial proportion of community-dwelling older adults experience suboptimal sleep quality. These individuals may exhibit symptoms such as non-restorative sleep, prolonged sleep latency, sleep maintenance difficulties, daytime functional impairment, and excessive daytime sleepiness. Given that participants were predominantly older adults from underserved communities, these findings underscore the necessity for contextually tailored interventions targeting modifiable determinants of poor sleep health in this population.

The results of the factor analyses provide empirical support for a three-factor structure of the SHI-C in the present sample. This structure is consistent with prior theoretical conceptualizations and previous empirical findings, suggesting that the dimensional organization of the construct is largely stable in this population. This finding was consistent with previous psychometric evaluation studies in the United States, France, and China (9, 13, 14). Most items had substantial factor loadings greater than 0.40, whereas the “unintentional doze” item showed a relatively lower loading in the sleep quality sub-index (0.216), and the “sleep medication” item showed a lower loading in the disordered sleep sub-index (0.150). Interestingly, the low factor loading of “sleep medication” was consistent with findings from U.S. adults (9), but differed from those reported for adults in France (13), Chinese college students (14) and Chinese pregnant women (15). This discrepancy likely reflects the unique context of older adults. In this study, 71.50% of participants had chronic diseases. Sleep problems are often viewed as a normal part of aging or comorbidity of chronic diseases, rather than a condition requiring medication (44). Consequently, older adults may underreport sleep disturbances and often avoid sleep medication due to concerns about dependence, side effects, or a belief that sleep symptoms should be tolerated rather than treated. This results in a highly skewed response pattern (with most participants reporting no use), which reduces the item’s ability to differentiate between individuals in terms of sleep health. Similarly, the low loading of “unintentional doze” has also been observed across multiple cultural contexts (9, 13, 16). In older adults, daytime impairment often appears as persistent fatigue or cognitive slowing rather than discrete dozing. Sensory declines in this study (34.30% with vision loss, 30.70% with hearing loss) may further affect how drowsiness was perceived and reported. Although these items showed suboptimal loadings, they were retained based on expert consensus due to their theoretical relevance to sleep health assessment (9). Removing them could weaken the scale’s clinical utility and content validity. These results highlight that item functioning can vary across populations due to differences in health perceptions and symptom experience. They also suggest the value of contextual interpretation and support potential future refinements. For instance, rewording “unintentional doze” to better capture persistent daytime fatigue could improve how the scale reflects sleep health in older adults.

The SHI-C has demonstrated robust construct validity, including concurrent, convergent, and known-group validity. It showed high correlations with the PSQI and ISI, confirming the SHI-C’s concurrent validity, consistent with prior research (16). Moderate to strong correlations with the GDS-15 and weaker correlations with the ESS further supported its convergent validity. Consistent with recent studies (45, 46), the SHI-C reliably detected subtle changes in sleep health associated with depressive symptoms and daytime sleepiness in our study, both of which significantly impact sleep quality. Additionally, the SHI-C successfully distinguished older adults with and without chronic diseases, reinforcing its known-group validity and demonstrating its applicability in sleep health assessment, particularly in chronic disease management. Furthermore, the overall SHI-C score showed a meaningful pattern of correlations with key objective health criteria, as a negative correlation with comorbidity burden (aCCI) and a positive correlation with physical function (HGS). These results link better sleep health to lower disease burden and higher vitality in this geriatric population, demonstrating that the SHI-C captures aspects of sleep health that are concretely associated with broader health and functional outcomes.

The SHI-C also exhibited satisfactory reliability in this study. The internal consistency for both the overall index and the sleep quality sub-index exceeded 0.70, consistent with findings from diverse populations, including U.S. adults (9), and Chinese patients with degenerative spinal diseases (16). The internal consistency of the sleep duration sub-index was comparable to that of Chinese college students (Cronbach’s *α* = 0.63) (14), but much higher than that of the French study (Cronbach’s *α* = 0.13) (13). However, Cronbach’s α for the disordered sleep sub-index was slightly above 0.5, falling short of the recommended threshold. This result was modestly higher than a recent validation among Chinese college students (Cronbach’s *α* = 0.47) (14), but slightly lower than that from U. S. adults (Cronbach’s *α* = 0.63) (9) and the French validation study (Cronbach’s *α* = 0.55) (13). Despite this, the sub-index demonstrated clear criterion validity, with significantly negative correlations to PSQI, ISI, and ESS. This indicates it still captures relevant sleep-related impairment, consistent with reports that sleep health measures can remain valid even with modest internal consistency (14, 16). Three factors may explain the lower reliability. First, the sub-index contains only three items, which limits internal consistency. Second, a ceiling effect was observed on the Disordered Sleep scale (mean = 91.30, SD = 19.39), as most participants reported no diagnosed sleep disorder or medication use. This limited the variability of the total scores. Third, cultural norms and limited healthcare access in Xinjiang may lead older adults to underreport sleep problems or to view them as age-related, with the results that the discriminant validity of relevant items is compromised. Further refinement of this sub-index is warranted to enhance its psychometric properties in similar populations. Test–retest reliability analysis confirmed the consistency of the SHI-C across time. A two-month interval was used for the re-test analysis. Results indicated that sleep scores across the three sub-indexes remained stable over time. The overall ICC for test–retest reliability was 0.784, with sub-index ICC values ranging from 0.536 to 0.857, indicating good reliability of the SHI-C in older adults. In summary, while the SHI-C demonstrated adequate reliability, further research is warranted to strengthen the internal consistency of the disordered sleep sub-index.

Despite its contributions, this study has several limitations that should be acknowledged. Firstly, the cross-sectional design limits the evaluation of the SHI-C’s predictive validity, and the absence of objective sleep measures (e.g., actigraphy or polysomnography) limits verification of self-reported data. Secondly, the sample was recruited only from community-dwelling older adults in Xinjiang using convenience sampling, which may limit generalizability to other regions or settings. Thirdly, although we supplemented comparisons showing the retest subsample was representative of the baseline cohort, the two-month retest interval, chosen to balance recall effects and trait stability, may nonetheless capture natural sleep variations, yielding a conservative reliability estimate. In addition, certain key items, including “unintentional doze” and “sleep medication,” exhibited suboptimal factor loadings, which may stem from their mismatched relevance to this geriatric cohort’s unique sleep experiences and healthcare-seeking behaviors, potentially compromising the scale’s measurement accuracy for this specific population. Future studies should address these limitations by employing longitudinal designs, integrating objective sleep metrics, expanding the participant pool to include a wider range of populations, and considering further adaptation of item wording or content to enhance cultural and age-specific appropriateness. Addressing these constraints will be essential for validating the SHI-C and deepening our understanding of sleep health among community-dwelling older adults.

## 5 Conclusion

In summary, this study validates the SHI-C as a reliable and valid tool for assessing sleep health in community-dwelling older adults. It underscores the necessity of population-specific adaptation for sleep instruments and highlights the clinical potential of such tools for the timely detection and management of sleep disturbances. Future refinements, particularly to the disordered sleep sub-index, could further enhance its utility in promoting health equity and quality of life in aging populations.

## Data Availability

The raw data supporting the conclusions of this article will be made available by the authors, without undue reservation.
