# Supplementary material for: Validity and reliability of the sleep health index among community-dwelling older adults
Source: Front Public Health. 2026 Apr 22;14:1651175. doi: 10.3389/fpubh.2026.1651175 (PMC13143789; doi:10.3389/fpubh.2026.1651175)
Supplement: Supplementary file 1 [file Table_1.DOCX]

**Supplementary Table 1 Baseline characteristics and comparison of the total sample and the retest group.**

| Variables | Total Sample (N = 274)  Mean (SD) or n (%) | Retest Group  (n = 60) | Statistic  (t/χ²) | *p*-value  (Non-Retest vs. Retest) |
| --- | --- | --- | --- | --- |
| Age (years) | 67.61 (5.84) | 67.45 (6.20) | 0.194 | 0.846 |
| Sex (male) | 132 (48.18%) | 28 (46.67%) | 0.045 | 0.832 |
| Education |  |  |  |  |
| elementary school and below | 198 (72.26%) | 42 (70.00%) | 0.534 | 0.766 |
| middle school | 63 (23.00%) | 16 (26.67%) |  |  |
| High school and above | 13 (4.74%) | 2 (3.33%) |  |  |
| Marital status |  |  |  |  |
| Married | 174 (63.50%) | 39 (65.00%) | 0.048 | 0.827 |
| Single / Divorce / Separation | 100 (36.50%) | 21 (35.00%) |  |  |
| Chronic diseases |  |  |  |  |
| Yes | 196 (71.53%) | 41 (68.33%) | 0.245 | 0.621 |
| No | 78 (28.47%) | 19 (31.67%) |  |  |
| BMI (kg / m²) |  |  |  |  |
| Overweight (BMI ≥ 23kg / m²) | 174 (63.50%) | 34(56.67%) | 2.765 | 0.429 |
| Vision |  |  |  |  |
| Decline | 94 (34.31%) | 21 (35.00%) | 0.010 | 0.918 |
| Normal | 180 (65.69%) | 39 (65.00%) |  |  |
| Hearing |  |  |  |  |
| Decline | 84 (30.66%) | 14 (23.33%) | 1.273 | 0.259 |
| Normal | 190 (69.34%) | 46 (76.67%) |  |  |
| Self-assessed Health Status |  |  |  |  |
| Good | 94 (34.31%) | 20 (33.33%) | 0.535 | 0.765 |
| Average | 120 (43.80%) | 29 (48.33%) |  |  |
| Poor | 60 (21.89%) | 11 (18.34%) |  |  |
| SHI-C | 84.29 (12.58) | 85.18 (11.75) | -0.503 | 0.615 |
| Sleep duration | 91.43 (11.98) | 89.98 (13.69) | 0.823 | 0.411 |
| Sleep quality | 70.18 (21.98) | 71.05 (20.91) | -0.279 | 0.780 |
| Disordered sleep | 91.30 (19.39) | 94.47 (15.22) | -1.189 | 0.235 |
| PSQI | 8.08 (3.95) | 7.80 (3.70) | 0.510 | 0.611 |
| Poor sleep quality (PSQI > 5) | 198 (72.26%) | 46 (76.67%) | 0.485 | 0.486 |
| ISI | 6.51 (5.14) | 5.82 (4.53) | 0.004 | 0.997 |
| Insomnia (ISI > 9) | 67 (24.45%) | 14 (23.33%) | 0.034 | 0.855 |
| ESS | 2.87 (3.34) | 2.87 (3.05) | 0.961 | 0.337 |
| GDS-15 | 2.78 (2.53) | 2.45 (2.30) | 0.944 | 0.346 |
| 15-Item Geriatric Depression Scale (GDS-15 ≥ 5) | 63 (22.99%) | 10 (16.67%) | 1.153 | 0.283 |
| aCCI | 3.85 (2.35) | 3.67 (2.21) | 0.554 | 0.580 |
| HGS (kg) | 18.58 (7.64) | 17.49 (6.80) | 1.025 | 0.306 |

SHI-C, The Chinese Sleep Health Index; PSQI, Pittsburgh Sleep Quality Index; ISI, Insomnia Severity Index; ESS, Epworth Sleepiness Scale; GDS-15, 15-Item Geriatric Depression Scale; aCCI, Age-adjusted Charlson Comorbidity Index; HGS, Handgrip Strength.
